# Radiographic and Venographic Appearance of Healthy and Laminitic Feet in Amiata Donkeys

**DOI:** 10.3389/fvets.2020.601665

**Published:** 2020-12-21

**Authors:** Irene Nocera, Benedetta Aliboni, Liri Ben David, Luis Alfonso Gracia-Calvo, Micaela Sgorbini, Simonetta Citi

**Affiliations:** ^1^Department of Veterinary Science, Veterinary Teaching Hospital, University of Pisa, Pisa, Italy; ^2^Private Equine Practitioner, Tel Aviv, Israel; ^3^Faculty of Veterinary Science, Veterinary Teaching Hospital, University of Helsinki, Helsinki, Finland

**Keywords:** Amiata donkey, laminitis, radiology, venography, foot

## Abstract

**Introduction:** Laminitis is a debilitating disorder resulting in irreversible anatomical changes in the feet of equids. Assessing specific anatomical features through radiography and venography provides diagnostic and prognostic information. The reference ranges are well-established in horses, but not in donkeys. It is also uncertain as to whether these ranges can be applied to every donkey breed. The present study characterizes the radiological and venographic hoof anatomy of healthy feet of Amiata donkeys and defines the changes associated with severe and mild laminitis.

**Materials and Methods:** A total of 16 forefeet were evaluated in 8 Amiata jennies. The animals underwent musculoskeletal examination, Obel grading assessment and radiological evaluation. Based on clinical examination and radiographic findings, the forefeet were grouped as healthy, mild or severe laminitic feet, thus the digital venograms were performed according to the group definition.

**Results:** Radiology revealed 7/16 healthy, 4/16 mild laminitic, and 5/16 severe laminitic forefeet. Statistical analysis showed differences between the healthy and laminitic forefeet for the dorsal angle (*p* < 0.0001) and angle of solar aspect (*p* < 0.0001) of the distal phalanx, for deviation between dorsal aspect of distal phalanx and the hoof wall (*p* < 0.0001) and phalangeal rotation angle (*p* = 0.0032). Venography was abnormal in mild and severe laminitic forefeet. In particular, the vascularization was reduced or absent at the lamellar-circumflex junction dorsally, at the sub-lamellar vascular bed and at the circumflex veins. Coronary plexus vascularization was absent in severe laminitic forefeet.

**Discussion and Conclusions:** This study provides the radiological parameters for the assessment of healthy and laminitic forefeet of Amiata donkeys. The mild laminitic foot venogram showed decreased vascularization mainly on lamellar-circumflex junction and sub-lamellar vascular bed, in latero-medial views. The severe laminitic foot showed very poor or absent vascularization in multiple areas. The technique is easily applicable and provides diagnostic support in laminitis.

## Introduction

Laminitis is an extremely painful disease that compromises the integrity of the digital dermis and the normal biomechanics of the equine foot (1). It can result in lameness and alterations in hoof horn production and in digit positioning, leading to chronic and acute foot pain (2). Chronic and irreversible cases, where animal euthanasia is the only humane option, are not rare (1).

The pathophysiology of laminitis is still not completely understood. It has been defined as the failure of the attachment of the distal phalanx and the inner hoof wall (1). The most commonly accepted causes are inflammatory and septic response, insulin resistance, mechanical overloading, and failure of the normal vascular perfusion within the foot (3).

These mechanisms lead to significant anatomical changes (4). This is of clinical importance because the diagnostic assessment can be aimed at detecting and quantifying of the anatomical changes (4–7). Today, radiological assessments are considered of primary diagnostic importance and represent the “gold standard” for the diagnosis of laminitis (8).

Digital venogram assessments have been shown to be very important for the assessment of vascular perfusion and integrity in the horse (9–12). This diagnostic method has the potential to provide information on the status of the blood supply within the foot capsule, to assess disease severity, to develop treatment strategies, and to monitor the response to treatment (10). Digital venograms can also be performed in very painful cases, even if the horse is not able to move at all, thus preventing further mechanical damage to the integrity of the digit dermis (10).

There is little information on the radiological anatomy of donkey feet (13–15), and therefore there has been a tendency to apply an equine model to help in the diagnostic interpretation (4, 7).

Laminitis is currently an underestimated pathology in donkeys, despite being a common disease in this species, due to the lack of physiological data on the donkey digit (16).

Few studies have been conducted on evaluating the radiographic appearance of the donkey digit, either in healthy or laminitic animals (4, 14), and few studies have been carried out on the normal aspect of the venogram in healthy donkeys (13, 17) and with evidence of laminitis (18).

The aim of this study was to assess the radiographic and venographic parameters of healthy Amiata donkey feet, to define the changes associated with mild and severe laminitis and to compare the results with other donkey breeds and horses.

## Materials and Methods

### Study Group

A cohort of 8 Amiata donkeys belonging to the Regional Stud Farm of Tuscany and housed at the Veterinary Teaching Hospital, Department of Veterinary Sciences, University of Pisa were enrolled in this study. Approval to conduct this study was obtained from the ethical committee of the University of Pisa, according to the D. Lgs. 26/14 (Number 23/19).

All the animals enrolled were barren jennies used for reproduction purposes and were considered non-athlete. The animals were aged between 9 and 19 years (median age 13 years), the body weight (BW) ranged between 283 and 393 kg (median BW 342 kg), and the body condition score (BCS) ranged between 5 and 6.5/9 (median BCS 5.75/9). Jennies were housed in collective paddocks 24 h a day, fed with meadow hay *ad libitum* along with commercial equine feed in line with the NCR energy recommendations (19). All the subjects were barefoot and underwent periodical hoof trimming every 50/60 days.

None of the jennies had a previous history of foot-related problems. An orthopedic evaluation was performed on all the animals to assess the following clinical signs related to laminitis: stance and gait irregularities, soundness according to the Obel score (2), digital pulse amplitude, increased hoof temperature, presence of supra-coronary depression, and increased sensitivity to hoof testers.

Hooves were inspected visually to assess the presence of the following signs of laminitis: presence of converging hoof growth rings, deformity of hoof capsule shape, slipper foot conformation, flattening of the sole, and widening of the white line. Since no previous studies are available in the literature, the presence of converging rings was assessed according to a grading scale specifically designed for this study. The wall deformity was thus scored from 0 to 3 (Table 1).

**Table 1:** Grading of the wall deformity (20, 21).

	**Wall deformity scale**
Grade 0	No visible changes
Grade 1	Converging hooves growth rings involving 1/3 of the hoof wall
Grade 2	Converging hooves growth rings involving 2/3 of the hoof wall
Grade 3	Wall changes involving the entire hoof wall

### Radiographic Technique

For all the jennies enrolled in the present study, x-ray views were obtained for both forelimbs, for a total of 16 forefoot radiographs. Prior to radiography, the feet were thoroughly cleaned. All subjects were sedated with detomidine chloride (10 μg/Kg, IV) (Detogesic®, Zoetis Italia, Italy) and buthorphanol 0.025 mg/kg, IV (Nargesic®, ACME, Italy) (22). Baseline radiographs, dorso-palmar (DP) and latero-medial (LM) views, were taken. The jennies were then placed on wooden blocks for the feet (8 cm in height) (4), positioning both forelimbs in a way that the metacarpi were perpendicular to the ground and in close contact with the radiograph cassette to prevent any image magnification (23). Radiopaque barium paste was applied on the dorsal hoof wall at the midline, up to the palpable proximal coronary band (23).

All radiographs were obtained at a focal distance of 80 cm, with the beam focused midway between the dorsal and palmar aspect of the foot, and midway between the coronary band and the weight bearing border, in accordance with the literature (4). All radiographic procedures were performed using a portable machine (GIERTH HF100 M), with the following settings: exposure factors of 59 kV at 1.2 mAs, for a 100 mm wide hoof (adjusting 1kV according to 5 mm change in width) (4). One single experienced operator performed all the radiographs (IN). Radiographs were scanned and the digitized images were analyzed using commercial software (Horos^TM^–DICOM).

### Radiological Parameters

All the forefoot radiographs obtained were assessed in terms of the radiological parameters relevant to laminitis. These radiometric parameters and associated definition are shown in Table 2 (4, 14).

**Table 2:** Direct and derived radiological parameters relevant in laminitis, for the forefoot and their definitions, in latero-medial and dorso-palmar radiographic views [modified from (4)].

**Parameter**	**Definition**	**Method of determination**
**ANGULAR PARAMETERS OF THE FOOT**		
**Latero-medial radiographic view**		
S	Dorsal hoof wall angle	Angle subtended between the dorsal aspect of the hoof wall and the ground line direct parameter
Ts	Dorsal angle of the distal phalanx	Angle subtended between the dorsal aspect of the DP and the ground line Direct parameter
U	Angle of proximal phalanx	Angle subtended between the long axis of the PP and the ground line Direct parameter
C	Angle of middle phalanx	Angle subtended between the long axis of the MP and the ground line Direct parameter
SA	Angle of solar aspect of the distal phalanx	Angle subtended between the solear aspect of the DP and the ground line Direct parameter
PA (U-C)	Angle of pastern axis	Angular difference between long axis of the PP and MP Derived parameter.
HPA (U-S)	Angle of hoof pastern axis	Angular difference between the dorsal hoof wall angle and the long axis of the PP
H Ang (Ts-S)	Angular deviation between the dorsal aspect of the DP and dorsum of the hoof wall	Angular difference between dorsal aspect of the DP and the dorsal hoof wall angle Derived parameter
F Ang (C-Ts)	DIP rotation Angle	Angular difference between dorsal aspect of the DP and the long axis of the MP Derived parameter
R Ang (U-Ts)	Phalangeal rotation angle	Angular difference between dorsal aspect of the DP and the long axis of the PP Derived parameter
**LINEAR PARAMETERS**		
**Latero-medial radiographic view**		
D	Distal displacement of the distal phalanx	Perpendicular linear distance between the proximal limit of the hoof wall and the extensor process of the DP
MPL	Middle phalanx length	Liner measurement of long axis of the middle phalanx
IDA	Proximal integument depth of the dorsal aspect of the foot	Perpendicular linear distance between the dorsal aspect of the hoof wall and the dorsal surface of the DP, immediately distal to the distal limit of the extensor process
IDB	Distal integument depth of the dorsal aspect of the foot	Perpendicular linear distance between the dorsal aspect of the hoof wall and the dorsal surface of the DP proximal to the apex of the DP
IDM	Mid integument depth of the dorsal aspect of the foot	Perpendicular linear distance between the dorsal aspect of the hoof wall and the dorsal surface of the DP at the midpoint between the IDA and IDB measurement sites
IDR	IDA/IDB ratio	
**Dorso-palmar radiographic view**		
SL	Lateral sole thickness	Perpendicular linear distance between the lateral solar aspect of the DP and the ground
SM	Medial sole thickness	Perpendicular linear distance between the medial solar aspect of the DP and the ground
LHW	Lateral hoof wall thickness	Perpendicular linear distance between the distal lateral aspect of the DP and the lateral hoof wall
MHW	Medial hoof wall thickness	Perpendicular linear distance between the distal medial aspect of the DP and the medial hoof wall
**MORPHOMETRIC PARAMETERS**		
**Latero-medial radiographic view**		
PPCA	Proximal palmar cortex angle	Angle subtended between the proximal palmar cortex of the DP and the ground line
PPCL	Proximal palmar cortex length	Linear distance between the point of insertion of DDFT and the articular process of the navicular joint
PCL	Palmar cortex length	Linear distance between the apex of the DP and the articular process of the navicular joint
RA	Reflex angle of palmar cortex	Internal angle subtended between the proximal and distal palmar cortex of the DP
AA	Apex angle	Internal angle subtended between the distal palmar cortex and the dorsal aspect of the DP
Surface Convexity	Dorsal surface of the coffin bone convexity	Quality evaluation of the convexity of the parietal surface of the distal phalanx
Erosion	Osteolysis of Distal margin of the coffin bone	Quality evaluation of resorption and remodeling of bone at the dorsal solear margin of the distal phalanx
Distal Margin Lip	Remodeling of the distal margin of the coffin bone	Quality evaluation of new bone formation on the dorsal aspect of the toe of the distal phalanx

### Subgroup Definition and Inclusion Criteria for Venography Protocol

Based on the results obtained from clinical examination and radiographic evaluation, the feet were retrospectively divided into three groups, as previously reported (15, 24): (A) healthy foot (7/16), which is normal at clinical examination and radiographic parameters where within normal limits, (B) foot showing mild laminitic changes (4/16), namely Obel grade <1 and no hoof capsule changes and radiographic findings of distal phalanx rotation, and (C) foot showing severe laminitic changes (5/16), which is characterized by Obel grade >1, hoof capsule deformation, and radiographic findings of distal phalanx displacement and remodeling. The venography exams were performed on 3/7 healthy feet, 4/4 mild laminitic feet, and 5/5 severe laminitic feet.

### Venographic Technique

All the venographic exams were performed under sedation with detomidine chloride (10 μg/Kg, IV) (Detogesic®, Zoetis Italia, Italy) and buthorphanol 0.025 mg/kg, IV (Nargesic®, ACME, Italy) (22). The hair was clipped from the distal third of the metacarpus up to the coronary band. The low four-point nerve block was performed by injecting 3 ml of lidocaine perineural at each site (lidocaine 2%, Zoetis Italia, Italy) and a tourniquet was tightly wrapped slightly above the fetlock, using constant tension. The area above the later palmar digital vein was scrubbed and a 21G butterfly IV catheter (Terumo Italia Srl, Italy) with an extension tube line was placed.

A total of 20 ml of contrast agent (Iopamiro 300®, Bracco Imaging, Italia) was used for an average-sized foot and two different syringes were used to avoid excessive injection pressure, and thus to prevent any perivascular extravasation or wall vein damage (24). The first 10 ml contrast was injected with the foot in weight-bearing position, immediately afterwards the second 10 ml was injected while the limb was gently flexed, thus taking the weight off the foot (24).

After all the contrast has been injected, the butterfly catheter was left in place and the tube line was taped proximally to the limb, until the radiographs were performed (18). The latero-medial and dorso-palmar views were taken with the limb in weight bearing position, within 45 s of the injection (24).

Six areas were evaluated on the venogram image (Table 3), as previously reported in the horse (24): palmar digital vein (PDV), terminal arch (TA), circumflex vessels (CV), lamellar–circumflex junction (LCJ), sub-lamellar vascular bed (SLVB), and coronary plexus (CP).

**Table 3:** Venographic parameters relevant in laminitis, for the forefoot and their definitions, in LM and DP radiographic views [modified from (24)].

**Parameter**	**Definition**	**Quality evaluation of contrast distribution**
**VENOGRAPHIC PARAMETERS**		
**Latero-medial radiographic view**		
PDV	Palmar Digital Vein	Present—Altered—Absent
TA	Terminal Arch	Present—Altered—Absent
CV	Circumflex Vessels	Location of the Circumflex Vessels distal to the palmar rim of the distal phalanx
LCJ	Lamellar-Circumflex Junction	Normal—Mild—Folded—Void of Contrast
SLVB	Sublamellar Vascular Bed	Uniform Line—Rectangular Shape—Triangular Shape—Void of Contrast
CP	Coronary Plexus	Normal—Abnormal—Void of Contrast
**Parameter**	**Definition**	**Method of determination**
**Dorso-palmar radiographic view**		
PDV	Palmar Digital Vein	Present—Altered—Absent
TA	Terminal Arch	Present—Altered—Absent
CVM	Circumflex Vessels Medial	Location of the Medial Circumflex Vessels distal to the palmar rim of the distal phalanx
CVL	Circumflex Vessels Lateral	Location of the Lateral Circumflex Vessels distal to the palmar rim of the distal phalanx
LCJ	Lamellar-Circumflex Junction	Normal—Mild—Folded—Void of Contrast
SLVB	Sublamellar Vascular Bed	Uniform Line—Rectangular Shape—Triangular Shape—Void of Contrast
CPL	Coronary Plexus Lateral	Normal—Abnormal—Void of Contrast
CPM	Coronary Plexus Medial	Normal—Abnormal—Void of Contrast

These areas were examined both in LM and DP views for a quality evaluation of the contrast distribution (24).

### Statistical Analysis

Data were assessed for distribution. Since some parameters were normally distributed and others were not, we decided to express all the results as not Gaussian distributed. The results were reported as median and standard error, minimum and maximum values.

The Kruskal-Wallis-test for multiple comparisons was applied to verify differences between the three groups concerning the numerical data (angular and linear radiographic parameters, lameness and clinical parameters of hoof convergent rings), BW and BCS. The significance level was set at p <0.05. Statistical analysis was performed with GraphPad Prism v. 8.3.1 (GraphPad Software Inc., San Diego, CA, USA).

## Results

The results obtained from physical and external hoof examinations are reported in Table 4, according to the different subgroups. In particular, the severe laminitic feet group presented 4/6 physical parameters and 4/6 altered external hoof parameters, compared to the results obtained for the normal and mild groups.

**Table 4:** Average values obtained from clinical evaluation, reported according to the subgroups.

	**Lameness**	**Altered stance at rest**	**Obel grade**	**Digital pulse amplitude**	**Hoof temperature**	**Response to hoof tester**	**BCS**	**Weight (kg)**
**PHYSICAL EXAMINATION**								
Normal	0	No	0	No	Normal	Negative	5	289
Mild	0	No	0	No	Normal	±	5.5	322
Severe	0-3	No	1	No	Warm	Positive	6	316
	**Palpable depression at coronary band**	**Convergent hoof rings**	**Shape deformity**	**Dropped sole**	**Widening white line**	**Slipper foot conformation**		
**EXTERNAL HOOF CAPSULE**								
Normal	No	1.1	Yes	No	±	No		
Mild	No	0.5	Yes	±	Yes	No		
Severe	Yes	1.2	Yes	Yes	Yes	No		

*BCS, Body Condition Score*.

**Figure 1:**
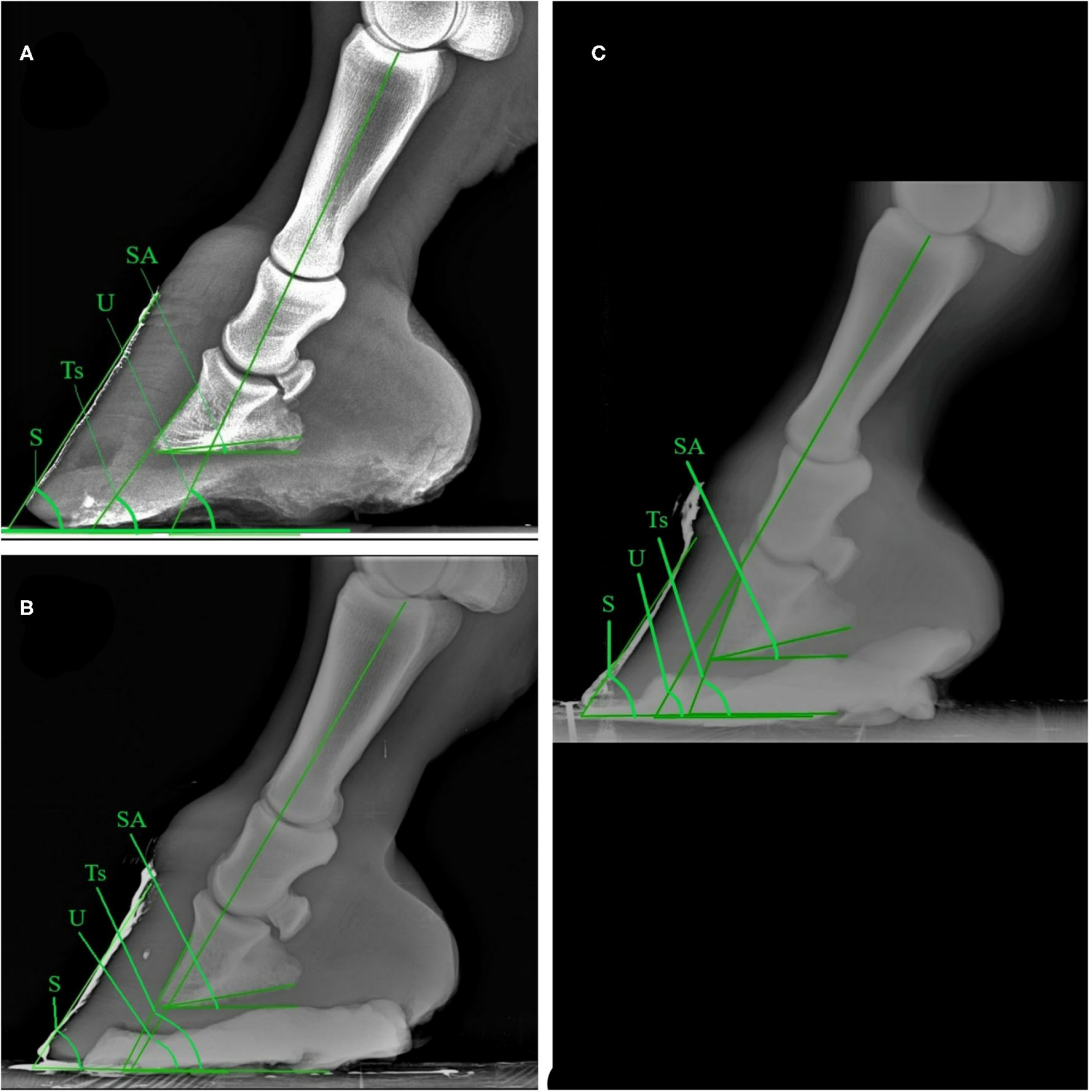
Latero-medial plain radiographic images of healthy **(A)**, mild **(B)**, and severe **(C)** laminitic donkey feet. SA, Angle of solar aspect of the distal phalanx; S, Dorsal hoof wall angle; Ts, Dorsal angle of the distal phalanx; U, Angle of proximal phalanx.

The results obtained for the angular, linear and morphometric radiological parameters relevant to the laminitis evaluation are shown in Tables 5–8, according to the different subgroups. Statistical analysis showed differences between the healthy and the severe laminitic donkeys in terms of Ts (*p* < 0.0001), SA (*p* < 0.0001), H ang (*p* < 0.0001) and R ang (*p* = 0.0032) (Figure 1). No statistical differences were obtained for the other parameters. According to the evaluation of the angular, linear and morphometric radiological parameters, only 1 out of 8 (12.5%) donkeys presented the left forefoot healthy and the right forefoot mild laminitic.

**Table 5:** Angular radiographic parameters from latero-medial view, expressed as degree, for the healthy, mild, and laminitic study groups.

**Angular parameters**																														
	**S**			**Ts**			**U**			**C**			**SA**			**PAxis (U-C)**			**HPAxis (U-S)**			**H Ang (Ts-S)**			**F Ang (C-Ts)**			**R Ang (U-Ts)**		
	**H**	**M**	**S**	**H**	**M**	**S**	**H**	**M**	**S**	**H**	**M**	**S**	**H**	**M**	**S**	**H**	**M**	**S**	**H**	**M**	**S**	**H**	**M**	**S**	**H**	**M**	**S**	**H**	**M**	**S**
Me	60.1	59.3	61.9	62.5	66.4	71.3	61.2	60.2	63.8	64.7	58.6	67.9	7.7	11.3	15.7	−0.8	0.0	3.5	2.2	0.1	8.2	2.5	7.6	11.1	2.2	−7.8	−5.0	0.6	−5.9	−7.2
m	59.0	58.2	55.6	60.6	66.1	68.3	58.4	50.0	61.3	53.1	50.3	58.1	6.1	10.1	14.4	−7.7	−0.4	−6.6	−2.1	−9.4	−0.7	0.1	4.1	6.5	−7.4	−17.1	−10.7	−3.5	−17.4	−10.
M	61.1	62.0	63.4	64.1	67.3	78.6	69.1	62.5	73.0	72.1	61.3	76.6	9.2	13.7	22.4	5.3	4.6	5.7	8.0	3.2	9.7	3.1	8.0	16.7	9.2	−4.7	−2.0	5.0	−4.1	−1.2

*Me, median; m, minimum values; M, maximum value; H, healthy group; M, mild laminitic group; S, severe laminitic group*.

**Table 6:** Linear radiographic parameters from latero-medial view, expressed in cm, for the healthy, mild, and laminitic study groups.

	**Linear parameters**																	
	**D**			**MPL**			**IDA**			**IDB**			**IDM**			**IDR**		
	**H**	**M**	**S**	**H**	**M**	**S**	**H**	**M**	**S**	**H**	**M**	**S**	**H**	**M**	**S**	**H**	**M**	**S**
Me	3.3	1.6	1.7	3.4	3.7	3.5	2.5	2.6	2.4	2.7	2.9	3.0	2.6	3.0	2.8	1.0	0.8	0.8
m	1.2	0.8	1.3	3.3	3.4	3.4	1.7	2.0	2.1	2.1	2.4	2.5	2.0	2.2	2.4	0.8	0.8	0.8
M	3.9	2.2	2.2	3.7	4.0	3.7	3.0	3.3	2.9	3.2	3.4	3.2	2.9	3.2	3.2	1.2	1.1	0.9

*Me, median; m, minimum values; M, maximum value; H, healthy group; M, mild laminitic group; S, severe laminitic group*.

**Table 7:** Morphometric radiographic parameters from latero-medial view, for the healthy, mild, and laminitic study groups.

	**Morphometric parameters**														
	**PPCA (mm)**			**PPCL (mm)**			**PCL (mm)**			**RA (degree)**			**AA (degree)**		
	**H**	**M**	**S**	**H**	**M**	**S**	**H**	**M**	**S**	**H**	**M**	**S**	**H**	**M**	**S**
Me	49.0	53.5	59.3	2.5	2.7	2.5	4.4	4.7	4.4	140.6	137.4	137.4	52.4	57.2	53.4
m	41.6	50.9	51.7	2.0	2.4	2.0	4.0	4.5	4.1	124.3	135.9	134.0	45.1	54.6	50.6
M	61.5	55.0	61.5	2.9	3.5	2.9	5.2	5.2	5.0	147.4	144.6	145.3	56.8	64.6	60.3

*Me, median; m, minimum values; M, maximum value; H, healthy group; M, mild laminitic group; S, severe laminitic group*.

**Table 8:** Linear radiographic parameters from dorso-palmar view for the healthy, mild, and laminitic study groups.

	**Linear parameters**											
	**SL (cm)**			**SM (cm)**			**LHW (cm)**			**MHW (cm)**		
	**H**	**M**	**S**	**H**	**M**	**S**	**H**	**M**	**S**	**H**	**M**	**S**
Me	2.5	2.0	2.1	2.4	2.3	1.9	2.0	2.2	2.0	1.8	1.7	1.8
m	1.9	1.5	1.8	1.7	2.1	1.8	1.6	1.2	1.4	1.5	1.5	1.2
M	2.9	2.8	2.7	3.1	2.7	2.9	2.4	2.7	2.8	2.0	1.8	2.0

*Me, median; m, minimum values; M, maximum value; H, healthy group; M, mild laminitic group; S, severe laminitic group*.

**Figure 2:**
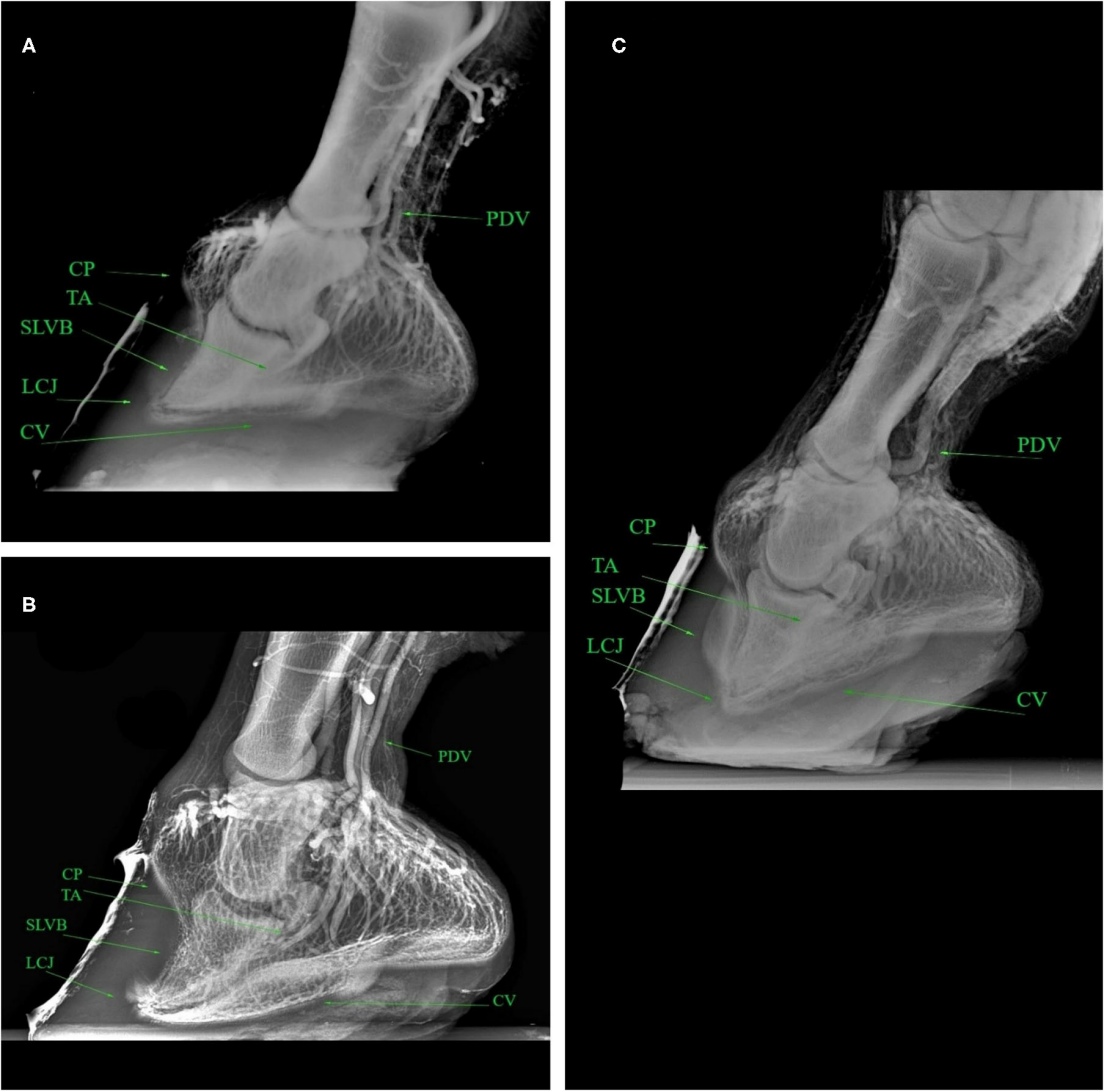
Latero-medial venograms of healthy **(A)**, mild **(B)**, severe **(C)** laminitic donkey feet. PDV, Palmar Digital Vein; CP, Coronary Plexus; TA, Terminal Arch; SLVB, Sublamellar Vascular Bed; LCJ, Lamellar-Circumflex Junction; CV, Circumflex Vessels.

**Figure 3:**
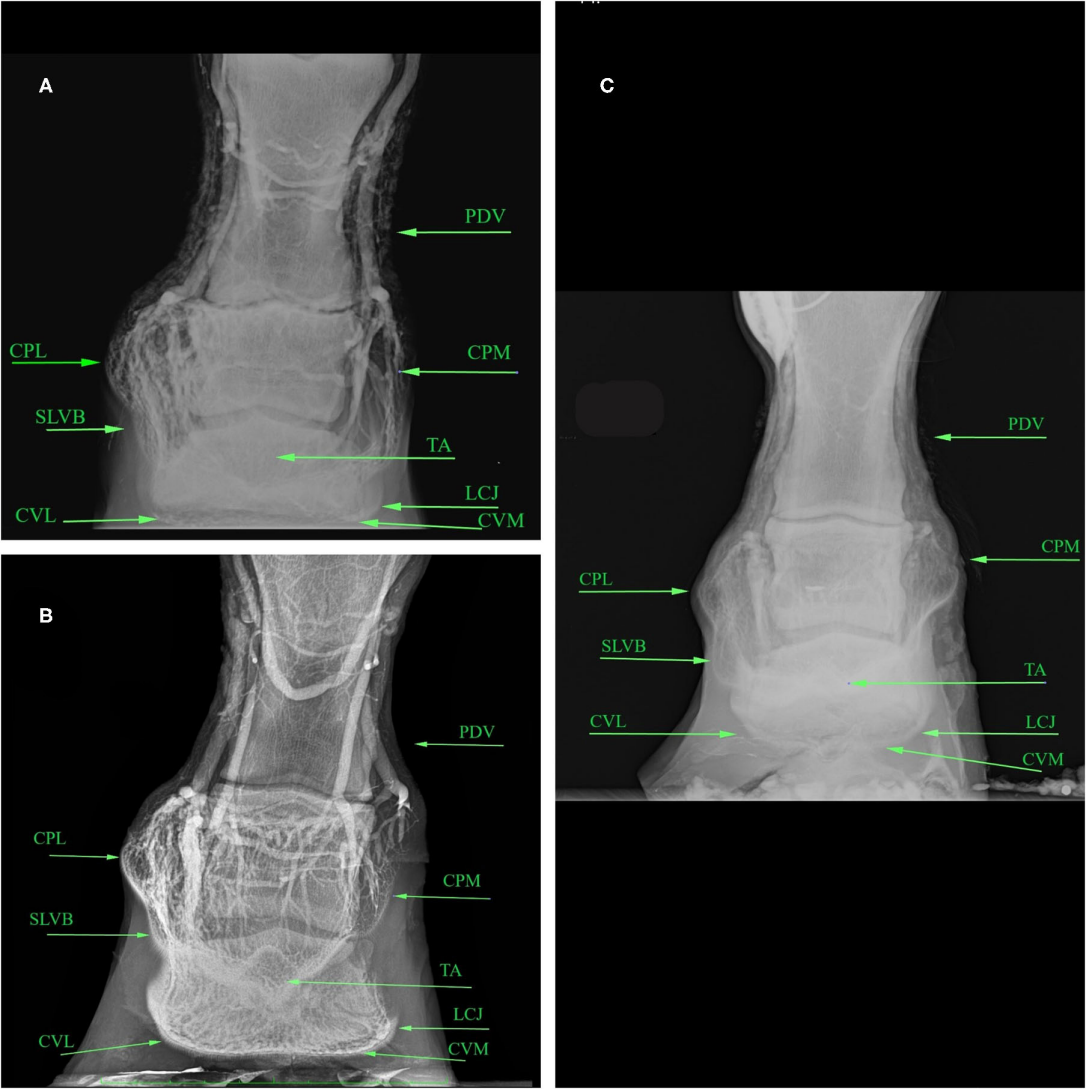
Dorso-palmar venograms of healthy **(A)**, mild **(B)**, laminitic **(C)** donkey feet. PDV, Palmar Digital Vein; CPL, Coronary Plexus Lateral; CPM, Coronary Plexus Medial; TA, Terminal Arch; SLVB, Sublamellar Vascular Bed; LCJ, Lamellar-Circumflex Junction; CVL, Circumflex Vessels Lateral; CVM, Circumflex Vessels Medial.

The results obtained from the evaluation of the venographic parameters are given in Table 9. Within the severe laminitic group, the main altered parameters both in the LM and DP were LCJ, SLVB and CP (both in the medial and lateral areas on DP view), compared to the healthy group. These parameters differ from the normal shape, with a total lack of contrast in the severe cases (5/16) (Figures 2, 3).

**Table 9:** Values obtained from the evaluation of venographic parameters, shown according to subgroups. Minimum and maximum values are reported for each parameter.

	**Normal group**	**Mild group**	**Severe group**
**LATERO-MEDIAL RADIOGRAPHIC VIEW**			
PDV	Present	Present	Present
TA	Present	Present	Present
CV (mm)	4.3 – 4.7	4.0 – 5.7	2.9 – 6.2
LCJ	Normal	Mild - Folded	Mild - Void of contrast
SLVB	Uniform Line	Rectangular to Triangular shape	Triangular Shape -Void of contrast
CP	Normal	Normal	Normal - Void of contrast
**DORSO-PALMAR RADIOGRAPHIC VIEW**			
PDV	Present	Present	Present
TA	Present	Present	Present
CVM (mm)	2.2–3.8	3.0–6.4	2.3–5.7
CVL (mm)	2.6–4.6	2.5–5.1	2.4–4.6
LCJ	Normal	Normal—Folded	Normal—Void of Contrast
SLVB	Uniform Line	Uniform line—Rectangular shape	Rectangular shape—Void of contrast
CPL	Normal	Normal	Normal—Abnormal
CPM	Normal	Normal	Normal—Void of Contrast

Finally, statistical differences were found for BCS (*p* = 0.0052), but not for BW (*p* = 0.0713) between severe laminitic vs. healthy group. In particular, the BCS was higher in the laminitic group.

## Discussion

The radiographic and venographic appearance of healthy and laminitic feet in donkeys were assessed in order to define the changes associated with mild and severe laminitis and to compare the results with other donkey breeds and horses.

Overall, we found statistical differences between healthy vs. severe laminitic donkeys for Ts, SA, H, and R ang, but not between mild vs. severe laminitic or healthy feet. No differences were found for other radiographic parameters. Thus, our results support the idea that the bone alignment and its relationship with the hoof capsule is relevant in the evaluation of laminitis in donkeys, in line with literature (4).

In our study, Ts and SA values differed significantly between severe laminitic and healthy group. In particular, Ts and SA values were higher in the severe laminitic group compared to the healthy group, in agreement with Collins et al. (4). Moreover, the Ts and SA values obtained in the severe laminitic group were comparable with the laminitic values reported by Collins et al. (4) in the horse, even if we found a narrower range of values for Ts (68.30–78.60° vs. 57–94°) (4). This difference might be related to the small population enrolled in this study, which could be considered a limitation to the present work. SA is commonly considered to be useful in the diagnosis and prognosis of laminitis in horses and the degree of rotation has been inversely related to the prognosis (25).

The statistically differences obtained in the H Ang values between healthy and severe laminitic groups were in line with the previous study by Collins et al. (4). Moreover, the laminitic H Ang values obtained in this study were comparable to what found both in donkeys (4) and horses (6) in previous studies. The H Ang value usually indicates the correct presence of the parallelism between the dorsal surface of the distal phalanx and the dorsal wall of the hoof capsule, both in donkeys (4, 14, 15) and horses (23, 26). The divergence in alignment supported by the increased H ang value might be indicative of dorsal distal phalanx rotation (23, 27).

In this work, the R Ang values statistically differ between healthy and severe laminitic groups, as also reported by Collins et al. (4). In particular, the laminitic R Ang values obtained in this study were lower than results found in previous studies performed both in donkeys (4) and horses (6). This finding might be related to the large variability in digit values reported in different studies performed in donkeys (4, 14, 15, 18). For this reason, even if the R Ang value is considered relevant for diagnosis of laminitis (4), it needs to be evaluated with caution.

The lack of differences between mild vs. severe laminitic feet or healthy feet for Ts, SA, H, and R ang might be related to the small number of animals included in the study and/or by the wide variability in digit values registered in donkey breeds (4, 14, 18) and, in particular, in Amiata donkeys (15).

We found 1/8 donkey presenting one healthy forefoot and the other one showing mild laminitic changes, in line with literature. In fact, laminitis may affect only one foot, if the causes are repeated trauma on the foot, abnormal distribution of the loading force and any other alteration of the normal gait (11, 23).

The PDV and TA values obtained from venographic studies in healthy donkeys were clearly evident, in agreement to literature (18). In this study, the PDV and TA values were evident also in mild and laminitic donkeys. These results are in line with previous studies in which the PDV and TA values were rarely altered in laminitic animals, even in severe cases (such as in DP distal displacement, infarcts, and thickening of the distal aspect of the deep digital flexor tendon) (10, 24).

In our study, the CV values, obtained both in the LM and DP views, showed wide ranges both in the mild and severe laminitic groups compared with the healthy one. To the best of authors' knowledge, the CV range has not been reported in healthy donkey feet yet. The results obtained for CV parameter in healthy donkeys in this study is comparable to what reported in a previous study (24). Sound horses showed a large variation in the normal appearance of the CVs and sole and may be affected by abnormalities others than laminitis (10, 24). Thus, our findings obtained in the mild and severe laminitic groups might be distorted by the coexistence of foot problems other than laminitis. CV should still be evaluated during a venogram laminitis assessment because an increase in solar depth and CV appearance may be related to successful treatment (10, 24).

On the other hand, our results for the LCJ and SLVB revealed strongly altered values in both the mild and severe laminitic groups compared with the healthy group, both in DP and LM views. Donkeys show a well-developed anastomosis in foot circulation compared to horses (18). In horses, LCJ and SLVB parameters were related to displacement of the DP, damage to the vessels secondary to displacement, and sub-lamellar edema (10, 24). It is possible that the particular extensive foot circulation in donkeys may lead these parameters get altered earlier compared to the other venogram parameters. Evaluation of LCJ and SLVB values may therefore be useful to promptly detect a potential anatomical vasculature alteration within the hoof.

Lastly, in our study CP parameters showed comparable results between the healthy and mild laminitic groups. On the other hand, the CP ranged from normal to void of contrast in the severe laminitic group. These findings may be explained considering that occasionally an inadequate volume of contrast creates a technical artifact, reducing the filling of CP, as reported in horses (24). Moreover, in severe laminitic horses, the CPs were found to be permanently distorted and so contrast may be reduced (10, 24).

As reported in literature, the presence of dermal pathology may be overlooked without venograms and the appropriate treatment may be delayed (24). According to this, our results seem to suggest that, also in donkeys as in horses, the venogram could detect mild laminitis change within the foot earlier than radiogram. In our study, the venogram was useful in demonstrate vascular and dermal integrity. It could be an important tool for the assessment of severity of the disease and the development of the treatment strategies also for donkeys.

This study has some limitations. The venogram appearance was evaluated in a small number of healthy feet, thus this might have led to a bias in the interpretation of results. Therefore, more studies needed to correctly establish the normal venogram appearance of healthy donkey feet in order to properly evaluate a pathological foot and to verify the reproducibility of the study.

## Data Availability Statement

The raw data supporting the conclusions of this article will be made available by the authors, without undue reservation.

## Ethics Statement

The animal study was reviewed and approved by the Ethical Committee of the University of Pisa, according to the D. Lgs. 26/14 (Number 23/19). Written informed consent was obtained from the owners for the participation of their animals in this study.

## Author Contributions

MS and SC conceived, designed, and supervised the project. IN, BA, and LB executed the experiment. IN, BA, and LG-C analyzed the data. MS executed the formal analysis. IN and MS had full access to all the data in the study and take responsibility for the integrity of the data, and the accuracy of the data analysis. All the authors interpreted the data, wrote and critically revised the manuscript for intellectual content, and approved the final version. All authors contributed to the article and approved the submitted version.

## Conflict of Interest

The authors declare that the research was conducted in the absence of any commercial or financial relationships that could be construed as a potential conflict of interest.
